# Medical Items

**Published:** 1895-03

**Authors:** 


### *MEDICAL ITEMS.*

We are told by Dr. Westmoreland, president of the Medical Association, that the prospects are encouraging for a good meeting at Savannah. A good program is being prepared, and we have Dr. Elliott's word for it that the Association will be well taken care of. We hope the meeting will be well attended. Don't forget the date—the 17th of April.

A Good Bill.—A bill has been introduced in the legislature of Iowa which provides that every patent medicine offered for sale in that State shall have a statement of the ingredients of the preparation printed on the wrapper. Failure to comply with this request is punishable by a maximum fine of $100, or six months in the penitentiary.—*Med. Bulletin.*

The Atlanta and West Point Railroad is the first road in the State to supply its trains with surgical appliances. Boxes have been placed in every baggage car and in every freight caboose, containing antiseptic dressings, bandages, tourniquet, splints, plaster of Paris, etc. This wise provision might be adopted by all roads with great advantage and comfort to passengers.

Dr. Wm. Waugh, in *Medical World*, says: Talk as you will, there is nothing like the knowledge a man gets by sitting down by his patient's bedside and watching him. I would rather put my faith in a man who studies his cases, than in one who injects drugs into rats and then jumps at the conclusion that the results he obtains are identical with those produced by the same drug in man.—*Ex.*

A bill has been introduced in the United States Senate, "to regulate the practice of medicine and surgery, to license physicians and surgeons, and to punish persons violating the provisions thereof, in the District of Columbia." The bill proposes to create three boards of examiners, regular, homeopathic, and eclectic. The bill deserves to pass, in spite of certain objectionable features, which will greatly diminish its effectiveness.

---

The twentieth annual meeting of the American Academy of Medicine will be held in one of the buildings of the Johns Hopkins University, Baltimore, on Saturday, May 4, and on Monday, May 6, 1895. The "Headquarters" of the Fellows of the Academy and the meetings of the council will be at the "Stafford." This meeting is held just prior to that of the American Medical Association, so that those who wish may attend both. The president of the American Academy is Dr. J. McF. Gaston, of Atlanta; secretary, Dr. Charles McIntire, Easton, Pa.

---

Here is another new medical journal, the *General Practitioner*. And from St. Louis, too. It admits in its salutatory or apology for existence that the world does not seem to be hungry or thirsty for a new journal; and it does not come to fill any "aching void" or "long-felt want." Modest, certainly. Its appearance, therefore, seems to be entirely superfluous. The position which it takes in opposition to the movement for an examining board in Missouri should damn it in the estimation of all respectable practitioners. A journal which begins life under such auspices deserves to go down to speedy oblivion.

---

Already dispute has begun over the question of priority in the use of serum therapy. It appears that Behring, Kitasato, Roux, etc., have several rival claimants for this honor. Professor Ferran, of Barcelona, has entered his claim, basing it on a published paper (April, 1890), entitled "Experimental Vaccination against the Diphtheria Poison." Professor Babes, of Buda Pesth, says that he and Dr. Lepp anticipated all others in applying the same principle to hydrophobia in 1889. The claim for America has been made by Dr. Nuttall, late of Johns Hopkins University. But some genius has discovered that this principle was familiar to Mithridates, the distinguished King of Pontus, many years before the above gentlemen flourished. Such is fame.

